# Community seroprevalence survey for yaws and trachoma in the Western Division of Fiji

**DOI:** 10.1093/trstmh/trw069

**Published:** 2016-12-09

**Authors:** Naomi Cocks, Merelesita Rainima-Qaniuci, Chelsea Yalen, Colin Macleod, Apisalome Nakolinivalu, Stephanie Migchelsen, Chrissy h. Roberts, Robert Butcher, Mike Kama, David Mabey, Michael Marks

**Affiliations:** a Clinical Research Department, Faculty of Infectious and Tropical Diseases, London School of Hygiene & Tropical Medicine, Keppel Street, London WC1E 7HT, UK; b World Health Organization, Fiji Country Office, Fiji; c Department of Dermatology, Tamavua Twomey Hospital, Fiji; d Fiji Centre for Communicable Disease, Ministry of Health, Suva, Fiji; e Hospital for Tropical Diseases, University College London Hospitals NHS Trust, LondonWC1E 6JB, UK

**Keywords:** Fiji, Neglected tropical diseases, Scabies, Trachoma, Yaws

## Abstract

**Background:**

Both yaws and trachoma are endemic in several countries in the Pacific. In co-endemic countries there may be potential synergies between both control programmes.

**Methods:**

We undertook a cluster randomised trachoma and yaws seroprevalence survey of children in the Western Division of Fiji. Children were examined for skin lesions consistent with active yaws. A dried blood spot was collected which was tested using the *Treponema pallidum* particle agglutination (TPPA) test and an ELISA to detect antibodies against Pgp3.

**Results:**

A total of 607 children from 305 households across 23 villages were recruited into the survey. On skin examination, no child had clinical evidence of yaws, and the TPPA assay was negative in all children (0%, 95% CI 0.0–0.6). The seroprevalence of Pgp3 antibodies was 20.9% (95% CI 17.8–24.6%).

**Discussion:**

In this study there was neither clinical nor serological evidence that transmission of yaws was ongoing. The Pgp3 seroprevalence pattern was consistent with either low level transmission of ocular *Chlamydia trachomatis* or exposure to *C. trachomatis* in the birth canal which is consistent with a survey conducted in the same region in 2013. These data suggest neither yaws nor ocular chlamydia infection are a significant public health problem in the Western Division of Fiji.

## Introduction

Yaws, caused by *Treponema pallidum* subsp*. pertenue*, is endemic in several countries in the Pacific.^1^ Trachoma, caused by ocular infection with *Chlamydia trachomatis*, is the leading infectious cause of blindness and is also endemic in the Pacific.^2,3^ Both diseases are classified as neglected tropical diseases (NTDs) by WHO. WHO has targets for both the global eradication of yaws and elimination of blinding trachoma as a public health problem by 2020.^4,5^ Central to the strategy for both of these targets is the use of community mass treatment with azithromycin, which is an effective therapy for both organisms. In countries where both yaws and trachoma are endemic there may be potential synergies between the control programmes at multiple levels including survey, intervention and surveillance.^6,7^

Of the countries where yaws remains endemic, three of the most high-burden countries are in the Pacific, including Papua New Guinea, the Solomon Islands and Vanuatu.^8^ Yaws has previously been reported in many other countries in the Pacific, including Fiji, but there are no current data from Fiji on the prevalence of active disease or the seroprevalence of infection.^9^ Accurate data on both clinical and serological prevalence of yaws in Fiji are needed to guide programmatic decision making.

Trachoma has been reported to be endemic in a number of countries in the Pacific, although population-based mapping studies have suggested a relatively low number of cases of trichiasis the late, blinding, stage of the disease – in the region. A recent survey completed in the Western Division of Fiji reported a low prevalence of ocular infection with *C. trachomatis* among children, and similar findings have been reported from other Pacific countries, including the Solomon Islands.^10^

Serology has recently emerged as a potential surveillance tool for trachoma programmes. Current measures of ocular infection (directly by PCR, or indirectly by clinical examination) provide only a cross-sectional snapshot of community prevalence, without necessarily giving any information about the changing exposure in a population over time. For trachoma, antibodies to Pgp3 are thought to be a long-lived and specific marker for prior *C. trachomatis* exposure.^11,12^ Serology is also a major tool in surveillance for yaws. Treponemal antibodies persist for life and cannot distinguish between yaws and syphilis. A prevalence of treponemal antibodies greater than 1% amongst children has been suggested as a possible trigger for more detailed yaws mapping studies.^13^

The collection of dried blood spot samples in prevalence surveys have recently emerged as a practical tool to allow collection and storage of a large number of finger-prick blood samples from which the seroprevalence of several infections can be assessed. This has notable advantages in facilitating integrated NTD mapping and surveillance activities.^14,15^ Prior to this study implementation of the SAFE strategy for elimination of trachoma had not yet commenced in Fiji and there had not been any azithromycin mass drug administration conducted in the country. We conducted an integrated survey to assess the seroprevalence of antibodies to *T. pallidum* and *C. trachomatis* in order to evaluate the need for further interventions for these NTDs in Fiji.

## Methods

We conducted a population-based cluster-randomised survey in the Western Division of Fiji in July and August 2015, where we had previously documented a low prevalence of both clinical signs of active and ocular infection with *C. trachomatis.*^2^ No mass distribution of azithromycin for trachoma or yaws has previously been carried out in this region, nor any recent penicillin-based mass treatment campaigns for yaws, to the best of the authors’ knowledge. Data are reported in line with the STROBE guidelines for cross-sectional surveys (Supplementary File 1). Study data are available in Supplementary File 2.

### Survey methodology

This was a two-stage cluster-randomised survey. Each cluster consisted of a single village. Villages were selected randomly, using probability proportional to size sampling, from a list of all communities in the Western Division of Fiji. On the day on the survey, in collaboration with local leaders, a list of all households was enumerated and 30 households selected using simple random sampling. All children aged 1–14 at sampled households were eligible for inclusion.

### Data collection

A nurse was trained in standardised dermatology. Examinations included the head and neck, limbs and trunk but excluded the genitals and buttocks. Individual-level data were collected on age, gender, and the presence or absence of skin lesions. For each skin lesion we recorded the location, clinical appearance and whether the lesion was consistent with yaws or an alternative diagnosis. A clinical case of yaws was defined on the basis of typical clinical findings of papillomatous or chronic, painless ulcerative skin lesions and using the WHO pictorial guide to yaws.^16^ The clinical diagnosis of scabies was based on features including morphology (burrows, papules, nodules, vesicles) and body distribution of rash; presence of pruritus on history or evidence of excoriation; contact history with individuals with a similar rash and itch; and consideration of differential diagnoses. The distribution of scabies lesions was noted using nine pre-defined body regions. Scabies severity was classified by the number of lesions present as mild (≤10 lesions), moderate (11–49 lesions) or severe (≥50 lesions or crusted scabies).

Active impetigo was diagnosed on the basis of discrete papular, pustular or ulcerative lesions with associated erythema, crusting, bullae or frank pus. Inactive impetigo was diagnosed by the presence of discrete, non-confluent healed superficial skin lesions. Severity of active impetigo was classified as very mild (≤5 lesions), mild (6–10 lesions), moderate (11–49 lesions) or severe (≥50 lesions).

All data were entered directly into Android smartphones using the OpenDataKit software package.^17^ Individuals with a skin condition requiring treatment were referred to the local health clinic where they were treated free of charge in line with Ministry of Health guidelines.

A finger-prick blood sample was collected onto a filter paper from all children regardless of clinical features. Filter papers were air-dried and stored in a sealed bag with a desiccant sachet. In individuals with ulcerative or papillomatous skin lesions consistent with yaws we also collected a swab sample of lesion exudate. Exudate was transferred to a FTA Elute Micro Card (GE Healthcare, Chalfont St Giles, UK) using three firm side-to-side motions of the swab across the card. Each card was placed in its own re-sealable plastic packet with an individual desiccant sachet. All samples were transferred to Lautoka Hospital and frozen at −20°C and then shipped to the London School of Hygiene & Tropical Medicine (LSHTM), London, UK for testing.

### Laboratory testing

Blood samples were tested at LSHTM. All laboratory testing was performed by individuals masked to the clinical findings. For yaws, a single extension of each filter paper sample was eluted as previously described and the elute tested using the *T. pallidum* particle agglutination test (TPPA; Mast Diagnostics, Bootle, UK).^18^ For trachoma, a separate extension of each filter paper was tested using an ELISA for Pgp3 developed by the US Centres for Disease Control (Diana Martin, personal communication). Dried blood spots were eluted into 250 µL of 1× phosphate-buffered saline (PBS) with 2.5% weight-for-volume milk powder plus 0.3% volume-for-volume tween 20 (PBST-milk). Immunolon 2HB plates were coated overnight with 50 ng per well glutathione S-transferase (GST)-tagged Pgp3 then blocked for 1 hour with PBST. Plates were bound for 2 hours with 50 µL of the elution mixture, then incubated at room temperature with rabbit anti-IgG for 1 hour. Plates were then incubated in the dark for 10 minutes with tetramethylbenzidine (TMB) and the absorbance read at 450 nm. Plates were washed four times with PBST between steps. A series of positive serum diluted in negative serum was run on each plate. Plates were re-run if the mean of each point in the dilution series fell more than 20% outside of pre-determined normal range. All specimens on the plate were normalised to a 20% dilution of presumed-positive material as that typically gave optical density (OD) readings around 1.

### Statistical analysis

We calculated the clinical prevalence of yaws and other common skin infections. A positive cut-off value for the Pgp3 ELISA was defined using a mixtures model.^19^ For the purposes of analysis we grouped age into 1–5 years, 6–10 years and 11–14 years of age. We categorised ethnicity as iTaukei, Indo-Fijian (the predominant ethnic groups in Fiji) or other. We calculated the seroprevalence of antibodies against *T. pallidum* and *C. trachomatis.* Random effects logistic regression was used to assess associations between age, sex, ethnicity and gender with both clinical findings and seroprevalence data controlling for the effect of clustering at household and village-level. Analysis was conducted in STATA 13.1 (StataCorp LP, College Station, TX, USA) and R 3.2.3 (the R Project for Statistical Computing).^20^

### Sample size

We calculated that a sample size of 1005 children was needed to detect a seroprevalence of both treponemal antibodies and Pgp3 antibodies of 5% in 1–14 year-olds with a degree of precision of ±2%, a design effect of 2, and a 10% non-response rate. Based on the Fiji national census we calculated that 30 households in each of 30 villages would be sufficient to reach this sample size.

### Ethical approval

Written informed consent was obtained from each participating child's parent or guardian by a member of staff fluent in the local dialect. Assent was obtained from all children with a signature or thumb print. Ethical approval for the study was granted by the ethics committees of the Fiji Ministry of Health (2015.65.WES) and LSHTM (10359).

## Results

A total of 607 children from 305 households across 23 villages were recruited into the survey. The median age was 6 years (IQR 4–9), 312 children were male (51.4%). There were 493 individuals of iTaukei descent (81.2%), 91 individuals were Indo-Fijian (15.0%) and 23 children were of another ethnicity (3.8%).

**Figure 1.**
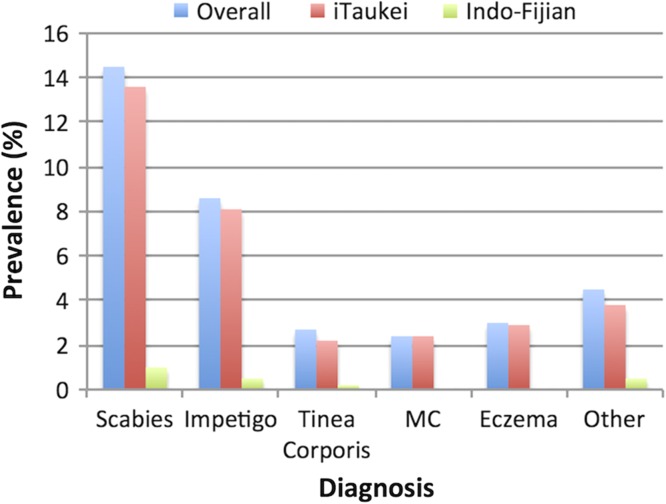
Clinical prevalence of common skin infections.

On examination 184 children (30.3%) had at least one skin lesion. No skin lesions were considered to be clinically consistent with yaws. The most common skin lesions noted were scabies without secondary bacterial infection (n=59, 9.7%), scabies with bacterial skin infection (n=31, 5.1%) and impetigo without underlying scabies (n=24, 3.9%). Scabies was more common amongst children of iTaukei ethnicity (OR 2.9, p=0.015) and was the major risk factor for the presence of impetigo (OR 10.30, p<0.0001) with a population attributable fraction of 51.2%. Eczema (n=18, 2.9%), tinea corporis (n=16, 2.6%) and molluscum contagiosum (n=14, 2.3%) were also common (Figure 1).

**Figure 2.**
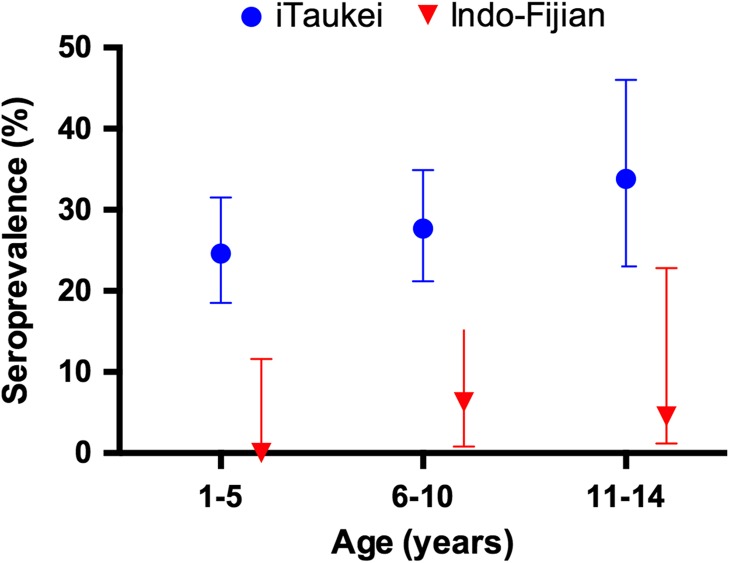
Seroprevalence of Pgp3 antibodies by age and ethnicity. Data are seroprevalence and 95% CIs. Only data for iTaukei and Indo-Fijian children are shown due to the small number of children of other ethnicities.

Dried blood spots were collected from 593 children (96.7%). The TPPA was negative in all children enrolled in this study (95% CI 0–0.6%). Because no yaws-like lesions were identified no swabs for PCR were collected. Using the mixtures model an OD threshold of 0.72 was defined for the Pgp3 assay. Based on this cut-off the overall seroprevalence of Pgp3 antibodies was 20.9%. Seroprevalence increased with age from 15% in children aged 1 year to 22% amongst children aged 14 years. In univariate analysis both age and ethnicity were significantly associated with a positive Pgp3 ELISA (p=0.043 and p<0.001 respectively) but there was no association with gender (p=0.71). In multivariable analysis iTaukei ethnicity was associated with a significantly increased risk of Pgp3 seropositivity (aOR 9.82, 95% CI 2.94–32.79, p<0.001) (Table 1 and Figure 2).

**Table 1.** Factors associated with Pgp3 antibody positivity

Variable	Indicative prevalence data	Adjusted odds ratio^a^ (95% CI)	p-value^b^
Age (years)			
1–5 (n=267)	16.9%	1	p=0.0133
6–10 (n=218)	23.3%	1.83 (1.10–3.02)	
11–14 (n=99)	25.2%	2.20 (1.17–4.15)	
Ethnicity			
Indo–Fijian (n=86)	3.5%	1	p<0.0001
iTaukei (n=476)	24.8%	10.88 (3.12–37.92)	
Other (n=22)	9.1%	2.89 (0.39–21.05)	
Gender			
Male (n=308)	20.5%	1	NS
Female (n=276)	21.7%	0.89 (0.57–1.38)	

NS: not significant.

^a^ All odds ratios are adjusted for age, gender and ethnicity.

^b^ Likelihood ratio test.

## Discussion

We found no evidence that yaws was still endemic in the Western Division of Fiji. No children were identified with clinical evidence of yaws and treponemal antibody testing was negative in all children. As these tests reflect lifetime exposure to *T. pallidum* this represents strong evidence that transmission of yaws is not ongoing in the communities where this study took place. Approximately one-fifth of children in the study had evidence of exposure to *C. trachomatis.* In a previous study in the Western Division of Fiji we reported a trachomatous inflammation – follicular (TF) prevalence of 2.8% and an ocular chlamydia infection prevalence of 2.3%.^2^ These serology data are comparable to other settings where there is a low prevalence of both TF and ocular chlamydia infection.^21^ Steeply increasing age-specific anti-pgp3 prevalence in children aged between 1 and 10 years is thought to be indicative of intense transmission of ocular *C. trachomatis*; in this population the prevalence of anti-pgp3 antibodies was already high in the 1-year age group, suggesting the majority of seropositivity was acquired at in the first year of life.^12,21^ As Pgp3 is conserved between urogenital and ocular strains of *C. trachomatis* we cannot discount the possibility that the Pgp3 seroprevalence may reflect transmission in the birth canal as well as, or indeed more than, ocular transmission of chlamydia. The prevalence of urogenital *C. trachomatis* infection amongst pregnant women has previously been reported to be high in Fiji and community prevalence surveys in other Pacific countries have also reported high rates of urogenital *C. trachomatis*.^22,23^ In a study of pregnant women in Suva, Fiji, native Fijian women were found to have higher rates of urogenital infections, including *C. trachomatis,* than Indo-Fijian women.^24^ Based on the current data it is unclear whether ocular *C. trachomatis* infection or transmission in the birth canal is the major driver of Pgp3 seropositivity in these communities. These data highlight the potential challenges of using Pgp3 as a sero-marker for trachoma in communities where the prevalence of genital chlamydia infection is also high.

Skin problems were common in this study population with scabies and impetigo the most commonly identified conditions. These findings are consistent with a national scabies prevalence survey that was previously conducted in Fiji.^25^ As previously noted scabies was more common amongst iTaukei than indo-Fijians and was the major risk factor for impetigo.^25^ The absence of both chronic ulcerative lesions and papillomatous lesions consistent with yaws is supported by our serological data showing an absence of treponemal antibody positivity. Anecdotally, health care staff in the Western Division of Fiji reported that they did not see children with skin lesions consistent with yaws presenting to health care clinics.

Our study has a number of limitations. Whilst survey clusters were chosen in advance, we did not achieve our desired sample size because of logistical difficulties on the days of surveys of particular communities and the time limitations of the survey team. This may have limited our ability to detect rare cases of yaws amongst these communities. This is of particular importance as yaws is known to be highly focal even in regions where it is endemic. However the absence of any positive treponemal antibody tests in children enrolled in this study provides strong evidence that transmission is not ongoing in the communities included in this study. The appropriate methodology for confirming yaws elimination has not yet been agreed but it is likely that large-scale seroprevalence surveys will be required. The reduced sample size also reduced the statistical power to assess associations between demographic variables and Pgp3 antibody status. Secondly, the study was conducted in only a limited geographic region of Fiji. Further studies in other parts of the country should be undertaken. Finally we did not collect linked clinical data on active trachoma or ocular swabs for PCR during this survey. We had conducted a previous survey in this region of Fiji two years earlier where we have demonstrated a low prevalence of TF (2.8%) and ocular *C. trachomatis* infection (2.3%) amongst children^2^ and where no cases of trachomatous trichasis were seen.

There is a need for better data to inform programmatic decision making and integrated approaches to NTDs in the Pacific. Our study has confirmed that yaws and trachoma mapping activities can be integrated and that it may be possible to expand this to other NTDs such as scabies. There is also scope for cooperation between mass treatment programs, for example co-administration of ivermectin, albendazole and azithromycin, where more than one treatment is indicated and appropriate safety and efficacy data exist.^26^ Our data add to existing data suggesting that yaws is no longer a public health problem in Fiji and that trachoma is not likely to be one of the major causes of blindness in Fiji. Given the mixed findings of trachoma surveys conducted in Fiji early impact assessments should be conducted to guide Ministry of Health trachoma elimination activities.

## Supplementary data

Supplementary data are available at Transactions online (http://trstmh.oxfordjournals.org/).

## References

[1] Marks M, Solomon AW, Mabey DC Endemic treponemal diseases. Trans R Soc Trop Med Hyg 2014;108:601–7. 25157125 10.1093/trstmh/tru128 PMC4162659

[2] Macleod CK, Butcher R, Mudaliar U et al Low prevalence of ocular *Chlamydia trachomatis* infection and active trachoma in the Western Division of Fiji. PLoS Negl Trop Dis 2016;10:e0004798. 27404379 10.1371/journal.pntd.0004798 PMC4942140

[3] Sokana O, Macleod C, Jack K et al Mapping trachoma in The Solomon Islands – results from the Global Trachoma Mapping Project. Ophthalmic Epidemiol 2016;Forthcoming. 10.1080/09286586.2016.1238946 PMC5706973 27937043

[4] Emerson PM, Burton MJ, Solomon AW et al The SAFE strategy for trachoma control: using operational research for policy, and implementation. Bull World Health Organ 2006;84:613–9. 16917648 10.2471/blt.05.28696 PMC2627433

[5] WHO Eradication of yaws – the Morges Strategy Wkly Epidemiol Rec 2012;87:189–94. 24340400

[6] Marks M, Vahi V, Sokana O et al Impact of community mass treatment with azithromycin for trachoma elimination on the prevalence of yaws. PLoS Negl Trop Dis 2015;9:e0003988. 26241484 10.1371/journal.pntd.0003988 PMC4524711

[7] Solomon AW, Marks M, Martin DL et al Trachoma and yaws: common ground. PLoS Negl Trop Dis 2015;9:e0004071. 26633176 10.1371/journal.pntd.0004071 PMC4669168

[8] Mitjà O, Marks M, Konan DJP et al Global epidemiology of yaws: a systematic review. Lancet Glob Health 2015;3:e324–31. 26001576 10.1016/S2214-109X(15)00011-X PMC4696519

[9] WHO Global Health Observatory Data Repository. Status of endemicity for yaws. Data by country. Geneva: World Health Organization; 2014 http://apps.who.int/gho/data/node.main.NTDYAWSEND?lang=en [accessed 2 October 2016].

[10] Butcher RMR, Sokana O, Jack K et al Low prevalence of conjunctival infection with *Chlamydia trachomatis* in a treatment-naïve trachoma-endemic region of the Solomon Islands. PLoS Negl Trop Dis 2016;10:e0005051. 27603015 10.1371/journal.pntd.0004863 PMC5014345

[11] Goodhew EB, Priest JW, Moss DM et al CT694 and pgp3 as serological tools for monitoring trachoma programs. PLoS Negl Trop Dis 2012;6:e1873. 23133684 10.1371/journal.pntd.0001873 PMC3486877

[12] Martin DL, Bid R, Sandi F et al Serology for trachoma surveillance after cessation of mass drug administration. PLoS Negl Trop Dis 2015;9:e0003555. 25714363 10.1371/journal.pntd.0003555 PMC4340913

[13] WHO Yaws strategy development: report of a meeting, 27–28 October 2014, Atlanta, GA, USA. Geneva: World Health Organization; 2015 . http://apps.who.int/iris/handle/10665/170990 [accessed 8 October 2016].

[14] Solomon AW, Engels D, Bailey RL et al A diagnostics platform for the integrated mapping, monitoring, and surveillance of neglected tropical diseases: rationale and target product profiles. PLoS Negl Trop Dis 2012;6:e1746. 22860146 10.1371/journal.pntd.0001746 PMC3409112

[15] Lammie PJ, Moss DM, Goodhew EB et al Development of a new platform for neglected tropical disease surveillance. Int J Parasitol 2012;42:797–800. 22846784 10.1016/j.ijpara.2012.07.002

[16] WHO Yaws: recognition booklet for communities. Geneva: World Health Organization; 2012 http://apps.who.int/iris/bitstream/10665/75360/1/9789241504096_eng.pdf?ua=1 [accessed 8 October 2016].

[17] Pavluck A, Chu B, Mann Flueckiger R, Ottesen E Electronic data capture tools for global health programs: evolution of LINKS, an Android-, web-based system. PLoS Negl Trop Dis 2014;8:e2654. 24722343 10.1371/journal.pntd.0002654 PMC3983089

[18] Smit PW, Vlis T, van der Mabey D et al The development and validation of dried blood spots for external quality assurance of syphilis serology. BMC Infect Dis 2013;13:102. 23442198 10.1186/1471-2334-13-102 PMC3586363

[19] Parker RA, Erdman DD, Anderson LJ Use of mixture models in determining laboratory criterion for identification of seropositive individuals: application to parvovirus B19 serology. J Virol Methods 1990;27:135–44. 2156877 10.1016/0166-0934(90)90130-8

[20] Johnson P adaptivetau: Tau-leaping stochastic simulation. R package version 2.2. 2014 https://CRAN.R-project.org/package=adaptivetau [accessed 8 October 2016].

[21] Martin DL, Wiegand R, Goodhew B et al Serological measures of trachoma transmission intensity. Sci Rep 2015;5:18532 doi:10.1038/srep18532. 26687891 10.1038/srep18532 PMC4685243

[22] Cliffe SJ, Tabrizi S, Sullivan EA, Pacific Islands Second Generation HIV Surveillance Group Chlamydia in the Pacific region, the silent epidemic. Sex Transm Dis 2008;35:801–6. 18580823 10.1097/OLQ.0b013e318175d885

[23] Walsh MS, Hope E, Isaia L et al Prevalence of *Chlamydia trachomatis* infection in Samoan women aged 18 to 29 and assessment of possible risk factors: a community-based study. Trans R Soc Trop Med Hyg 2015;109:245–51. 25732755 10.1093/trstmh/trv014

[24] Gyaneshwar R, Nsanze H, Singh KP et al The prevalence of sexually transmitted disease agents in pregnant women in Suva. Aust N Z J Obstet Gynaecol 1987;27:213–5. 3124803 10.1111/j.1479-828x.1987.tb00989.x

[25] Romani L, Koroivueta J, Steer AC et al Scabies and impetigo prevalence and risk factors in Fiji: a national survey. PLoS Negl Trop Dis 2015;9:e0003452. 25738499 10.1371/journal.pntd.0003452 PMC4349858

[26] Coulibaly YI, Dicko I, Keita M et al A cluster randomized study of the safety of integrated treatment of trachoma and lymphatic filariasis in children and adults in Sikasso, Mali. PLoS Negl Trop Dis 2013;7:e2221. 23675549 10.1371/journal.pntd.0002221 PMC3649960

